# Evaluating a Conditional Cash Transfer Scheme in a Maternal Health Care Utilization Program Among Rural Pregnant Women in Mysore District, India

**DOI:** 10.1089/whr.2019.0021

**Published:** 2020-06-10

**Authors:** Sandra Kiplagat, Makella S. Coudray, Kavitha Ravi, Poornima Jayakrishna, Karl Krupp, Anjali Arun, Purnima Madhivanan

**Affiliations:** ^1^ Department of Epidemiology, Robert Stempel College of Public Health and Social Work, Florida International University, Miami, Florida, USA.; ^2^ Public Health Research Institute, Yadavgiri, Mysore, India.; ^3^ Department of Health Promotion Sciences, Mel and Enid Zuckerman College of Public Health, University of Arizona, Tucson, Arizona, USA.; ^4^ Division of Infectious Diseases, College of Medicine, University of Arizona, Tucson, Arizona, USA.; ^5^ Department of Family and Community Medicine, College of Medicine, University of Arizona, Tucson, Arizona, USA.

**Keywords:** conditional cash transfer, India, *Janani Suraksha Yojana*, pregnant, rural

## Abstract

***Background:*** According to the World Bank report in 2015, the maternal death rate in India was 174 per 100,000, which is among the highest in the world. The Indian Government launched the *Janani Suraksha Yojana* (JSY) conditional cash transfer program in 2005 to curb the adverse birth outcomes by promoting institutional delivery and providing antenatal care (ANC) services for pregnant women. This study evaluates the factors associated with JSY conditional cash transfer program in rural Mysore, India.

***Methods:*** Between 2011 and 2014, a prospective cohort study was conducted to examine the feasibility and acceptability of integrated ANC and HIV testing using mobile clinics in rural Mysore. Pregnant women in the *Mysore Taluk* provided an informed consent and answered an interviewer-administered questionnaire in local language, *Kannada*. All women underwent routine ANC services and were followed-up immediately after delivery, and 6 months and 12 months after delivery. Binary logistic regression was performed to identify factors associated with JSY benefits.

***Results:*** The mean age of the 1,806 mothers was 21.2 ± 2.2 years and 58.9% of the mothers had primary education. Nearly half (51.6%) of the women reported having received JSY benefits. Factors associated with receiving JSY benefits included pregnant woman's partner not having any formal education (adjusted odds ratio [AOR]: 1.35; 95% confidence interval [CI]: 1.01–1.80), having income ≤4,000 Indian Rupees (AOR: 1.47; 95% CI: 1.04–2.09), rare visits (once in 3 months visit) with Accredited Social Health Activists (AOR: 3.55; 95% CI: 1.55–8.51), and delivery in a public institution (AOR: 1.23; 95% CI: 1.01–1.51).

***Conclusions:*** While JSY has been operational in India since 2005, there continue to remain major gaps in the receipt of JSY services in rural India. Future interventions should include targeted services and expansion of JSY scheme, specifically among rural pregnant women, who are most at need of these services.

## Introduction

Maternal health care access remains a significant public health challenge in India. According to the World Bank report in 2015, the maternal death rate was 174 per 100,000 (Ref.^1^). Majority of these deaths have been attributed to poor in-facility birth coverage, unskilled birth attendance, and inaccessibility to quality obstetric care.^2,3^ The Indian Government launched the *Janani Suraksha Yojana* (JSY-translated as the Safe Motherhood Scheme) conditional cash transfer program in 2005 to curb adverse birth outcomes by promoting institutional delivery and providing antenatal services.^3,4^ JSY is a component of the National Rural Health Mission that aimed to provide cash assistance and minimize out-of-pocket expenditure of maternity care, including antenatal care (ANC), delivery, and postnatal care.^4,5^

JSY promotes ANC visits and institutional delivery by frontline health workers commonly referred to as accredited social health activists (ASHA).^4,6^ They provide ancillary services, including transportation, navigation of the health system, and referral services.^6,7^ The cash benefits vary based on low-performing and high-performing states established on the number of institutional deliveries before the implementation of JSY. Low-performing states had been characterized as poor in-facility birth coverage and health services, and financial assistance is provided to all women regardless of their socioeconomic status or caste, whereas high-performing states had a history of higher rates of institutional deliveries and improved health services.^8,9^

According to the Ministry of Health and Welfare, the government would provide 1,400 Indian Rupees (INR) (approximately U.S. Dollar [USD] 20.12) to women belonging in rural and 1,000 INR (approximately USD 14.37) in urban low-performing states, whereas those in high-performing states would receive 700 INR and 600 INR for rural and urban areas, respectively.^9,10^ In high-performing states, financial assistance is provided to only women issued with the below poverty line (BPL) card, and those who belonged to a scheduled caste or tribe,^6^ whereas cash payments are given to all women in low-performing states.

Since its inception more than a decade ago, it remains the largest conditional cash transfer program in the world serving up to 10 million pregnant women each year and has a budget of approximately USD 452 million.^4,9,11^ Although this program was established more than a decade ago, prior studies have reported lack of awareness among Indian pregnant women.^12^ Moreover, there have been inconsistencies observed by JSY benefits that have varied from state to state, but largely influenced by educational attainment, possession of BPL cards, and increased ANC.^6,13^ There have been very few studies that have explored the impact of JSY in rural communities. Majority of the studies have been large scale evaluating low-performing compared with high-performing states.^4,6,10,13^

Since JSY has been operational for >14 years in the state of Karnataka, it was found appropriate to evaluate how JSY performed in the Mysore Subdistrict. The government assigned Karnataka as a high-performing state with mothers residing in rural areas receiving 700 INR and mothers residing in urban areas receiving 600 INR. This would be among the first studies conducted in Mysore District. The aim of the study was to assess how JSY performed in rural Mysore, specifically examining the receipt of JSY benefits, utilization, and factors associated with JSY benefits.

## Methods

### Study setting

Based on the 2011 census, the Mysore District had a population of 3,001,027, of which 1,489,527 were females.^14^ Of the population, 58.5% resided in rural villages. The literacy rate among females (67.1%) was lower compared with males (78.5%).^14^ Approximately 87.7% of the Mysore population self-identified as Hindu, 9.7% as Muslim, and 1.3% as others.^14^ Scheduled caste comprised 17.9%, while scheduled tribe was 11% of the population. The languages commonly spoken in Mysore were Kannada (81.2%), Urdu (8.6%), and Telugu (3.3%). The labor workforce was mainly agricultural sector and the average per capita income was 95,591 INR equivalent to 1,374 USD.^14^ More specifically, the National Family Health Survey-4 indicated that only 31.4% of rural pregnant women in Karnataka received full ANC, which comprised four ANC visits, at least one tetanus toxoid injection, and daily iron folic acid tablets for 100 or more days.^15^ The maternal mortality ratio (MMR) in Karnataka remains the highest in South India without significant improvements comparing 2011–2013 and 2014–2016 years with MMR as 133 and 108 per 100,000 live births, respectively.^1^

### Study design

Between 2011 and 2014, a prospective cohort study, known as the Saving Children Improving Lives (SCIL) project was initiated in the rural Mysore Subdistrict, Karnataka. The SCIL project utilized mobile clinics to deliver integrated ANC and HIV testing with the support of community health workers. This study is a health care utilization analysis conducted from the SCIL project. Detailed methods for the SCIL project were described elsewhere by Kojima et al.^16^

### Compliance with ethical standards

#### Ethical approval and consent to participate

The protocol of the study was reviewed and approved by the Institutional Review Boards of Public Health Research Institute of India and Florida International University. Confidentiality of all participants was maintained using a study identification number on all study records. Permissions were obtained from the District AIDS Prevention and Control Officer, the District Health Officer, and the *Taluk* Medical Officer for Mysore District, before carrying out the study.

### Study participants

A total of 1,820 pregnant women living in Mysore District, received integrated ANC and HIV testing through the SCIL project. During the mobile clinic visit, interested pregnant women receiving clinical services were informed of the study and invited to participate in the study. Eligible participants were pregnant women, 18 years and older residing in select village for >6 months. If women met the eligibility criteria, they underwent an informed consent process in a private location. On completion of the informed consent process, participants completed an interviewer-administered questionnaire in the local language of *Kannada*. This comprised sociodemographic, current reproductive history, and clinical laboratory examinations for sexually-transmitted infections during pregnancy. The questionnaire comprised maternal and newborn infant health complications, birth outcomes, as well as utilization of health services after childbirth. All women enrolled in the study were followed-up immediately after delivery, and 6 and 12 months postdelivery.

### Outcome and explanatory variable selection

The main outcome of this regression analysis assessed the factors that influenced the receipt of JSY benefits reported as “yes” or “no” during and after pregnancy. The explanatory variables included education defined as “none” for no formal education, “primary” as 1–8 years of education, and “secondary” for those with 8 or more years of education. According to India's Ministry of Statistics and Programme Implementation, the monthly per capita income in India is ∼10,000 INR.^17^ With regard to total monthly household income, majority of the participants mainly relied on subsistence farming and so it was defined as low at ≤4,000 INR (approximately USD 57.9 with 1 USD = 69 INR during the period of data collection), middle as 4,000–10,000 INR (USD 144.90), and high as ≥10,000 INR. Since, the monthly per capita income was stratified into the respective categories, the cutoff for the poverty line was established as ≤4,000 INR. Belonging to a microeconomic group was labeled as “yes” or “no.”

The social support program and services offered to pregnant women by the government, including ASHA and auxiliary nurse midwives (ANM), was also assessed. The frequency of the ASHA visits during pregnancy and for ANC visits included regular visits (once a month), occasional visits (twice a month), rare visits (once in 3 months), and never (no visits at all). Moreover, ASHA's role to plan transport arrangements before delivery was assigned as yes or no. Low birth weight of the infant was defined as having birth weight <2,500 g at the time of delivery.

Vital status of the baby was defined as “living” or “dead” within 28 days of delivery. Place of childbirth was categorized as “home,” “public institution,” which included subcenter, primary health center, or district health hospital, and “private institution” as maternity or private nursing home. ANC visits have been defined as “≤4 ANC visits,” “5–9 ANC visits,” and “greater than 9 ANC visits.” Birth attendance was defined as individuals present during the delivery process and this was “traditional birth attendant,” “relative,” “auxiliary nurse midwife,” and “doctor/nurse.” All sociodemographic variables were self-reported by the study participants at baseline, and during follow-up. The pregnancy outcomes were assessed during follow-up and confirmed by the hospital discharge notes.

### Statistical analyses

Data were entered in a Microsoft Access database and then exported to Excel database to allow for data analysis (Microsoft Corporation, Redmond, WA). Normality tests and nonparametric tests were conducted for those variables that violated normal distribution assumptions. Descriptive analyses were conducted for both categorical and continuous variables. Frequencies and percentages were done for categorical variables, while mean and standard deviation were calculated for continuous variables. Chi-square analyses and analysis of variance were computed to identify any significant associations in the categorical and continuous variables conducted after normality tests. Variables with chi-squared tests with *p*-values ≤0.25 were included in the binary logistic regression. Binary logistic regression was performed to identify factors influencing receipt of JSY benefits and the explanatory variables. The measures of association included crude and adjusted odds ratio (AOR), and the associated 95% confidence intervals (CIs). The variables were selected *a priori* for the model based on previous literature.^12,18^ A two-tailed statistical significance test of *p* ≤ 0.05 was done for all the analyses. All analyses were performed using Statistical Package for the Social Sciences (SPSS) version 23 (SPSS, Inc., Chicago, IL).

## Results

### Demographic characteristics

Of the 1,820 women enrolled in the study, a total of 1,806 women were eligible for this analysis. Fourteen women (0.7%) were excluded due to incomplete data on the survey. The mean age of the mothers was 21.2 ± 2.2 years. The majority of the women had more than primary education (58.9% and 46.0% for the women and their spouses, respectively). The primary religion reported was Hindu (98.6%). More than a third (36.4%) earned ≤4,000 INR (approximately USD 56) monthly, and almost half belonged to a microeconomic self-help group (49.6%). Just over half of the women (56.2%) delivered at a maternity or private nursing institution, while the rest 42% delivered in a subcenter or primary health care facility or district hospital. Only 1.3% of the women delivered at home. The most common mode of delivery was vaginal (82.0%) and 60% of the women were primigravida. Births were commonly assisted by a doctor or nurse (86.0%) followed by ANM (12.0%). Nearly 13.5% of the women gave birth to low birth weight infants (<2,500 g).

### JSY beneficiaries

Nearly 50% (51.6%) of the women reported having received JSY benefits (Table 1). Approximately 52.1% and 38.9% had received JSY from ANM and ASHA, respectively (Table 2). Nearly 45.5% received 1,500 INR, while 15% received 1,000 INR, 37.7% received 700 INR, and 1.7% received 500 INR. Majority of them (64.9%) received the JSY benefits within 30 days of delivery.

**Table 1.** Sociodemographic Characteristics of Pregnant Women Attending Saving Children Improving Lives Mobile Clinics, in Rural Mysore, India (*N* = 1,806)

Variables	Receipt of JSY benefits		p
	No (n* = 874; 48.4%), *n (%)	Yes (n* = 932; 51.6%), *n (%)	
Sociodemographic factors			
Age (mean, SD) in years	21.4 ± 3.2	21.1 ± 3.1	0.044
Pregnant woman's education (in years)			0.219
No education	64 (7.3)	50 (5.4)	
Primary (1–8)	304 (34.8)	324 (34.8)	
More than primary (>8)	506 (57.9)	558 (59.9)	
Partner's education (in years)			0.038
No education	149 (17.0)	172 (18.5)	
Primary (1–8)	296 (33.9)	358 (38.4)	
More than primary (>8)	429 (49.1)	402 (43.1)	
Religion			0.968
Hindu	862 (98.6)	919 (98.6)	
Muslim/others	12 (1.4)	13 (1.4)	
Total monthly household income (in INR)			0.141
≤4,000	306 (35.0)	351 (37.7)	
4,001–10,000	463 (53.0)	494 (53.0)	
>10,000	105 (12.0)	87 (9.3)	
Microeconomic self-help group membership			0.080
No	459 (52.5)	451 (48.4)	
Yes	415 (47.5)	481 (51.6)	
Programs			
ASHA visits during pregnancy			<0.001
Regularly (once a month)	638 (73.0)	722 (77.5)	
Occasionally (once in 2 months)	73 (8.4)	51 (5.5)	
Rarely (once in 3 months)	9 (1.0)	33 (3.5)	
Never	154 (17.6)	126 (13.5)	
ASHA accompanying woman to her ANC visits			<0.001
Regularly (once a month)	410 (46.9)	448 (48.1)	
Occasionally (once in 2 months)	92 (10.5)	59 (6.3)	
Rarely (once in 3 months)	11 (1.3)	37 (4.0)	
Never	361 (41.3)	388 (41.6)	
ASHA arranged for ambulance during labor			<0.001
No	507 (58.0)	419 (45.0)	
Yes	367 (42.0)	513 (55.0)	
Pregnancy outcomes			
Birthweight			0.831
Low birth weight (<2,500 g)	114 (14.5)	130 (14.2)	
Normal birth weight (≥2,500 g)	670 (88.5)	787 (85.8)	
Vital status of the baby			<0.001
Died	90 (10.3)	13 (1.4)	
Living	784 (89.7)	919 (98.6)	
Antenatal checkups			0.021
≤4 antenatal checkups	25 (3.2)	14 (1.5)	
5–9 antenatal checkups	664 (84.6)	813 (88.5)	
10 and more antenatal visits	96 (12.2)	92 (10.0)	
Delivery place			0.015
Home	15 (1.9)	7 (0.8)	
Public institution	311 (39.7)	413 (44.9)	
Private institution	458 (58.4)	499 (54.3)	
Birth attendance			0.028
Traditional birth attendant	4 (0.5)	7 (0.8)	
Relative	10 (1.3)	2 (0.2)	
Auxillary nurse midwife	89 (11.4)	126 (13.7)	
Doctor/nurse	681 (86.9)	784 (85.3)	

Public institution: subcenter, primary health care, district health center. Private institution: maternity home/private nursing.

ANC, antenatal care; ASHA, accredited social health activists; INR, Indian Rupees; JSY, *Janani Suraksha Yojana*; SD, standard deviation.

**Table 2.** Characteristics of *Janani Suraksha Yojana* Recipients Among Saving Children Improving Lives Program Participants in Rural Mysore, India

Variables	Frequency (%)
JSY benefits	
Receipt of JSY benefits	
No	874 (48.4)
Yes	932 (51.6)
From whom were provided JSY benefits	
ASHA	363 (38.9)
*Anganwadi* teacher	65 (7.0)
ANM	486 (52.1)
Doctor	18 (1.9)
Receipt of JSY benefits (in INR)	
500	17 (1.8)
700	351 (37.7)
1,000	140 (15.0)
1,500	424 (45.5)
Time period for JSY benefit delivery	
Within 7 days	210 (22.5)
Between 7 and 30 days	103 (11.1)
After 30 days	605 (64.9)
After 1 year	14 (1.5)
Microeconomic self-help group membership	
No	451 (48.4)
Yes	481 (51.6)

ANM, auxiliary nurse midwives.

Table 1 shows bivariate analysis of JSY beneficiaries. Among those who received JSY, 59.9% had more than primary education, while 37.7% had a total monthly income of ≤4,000 INR. More than 50% (53.0%) of those who received JSY had an annual income of 4,000–10,000 INR. On the contrary, 35% of those who earned ≤4,000 INR did not receive any JSY benefits. Of those who earned ≤4,000 INR, nearly half (46.6%) did not receive any JSY benefits. Approximately 13.5% of the participants who received JSY reported never having had visits from an ASHA worker during the course of their pregnancy. The proportion of women having ASHA accompany them regularly (once a month) to their ANC visits was significantly higher among those who received JSY compared with those without JSY benefits. The women were also significantly less likely to have delivered at home if the woman had received JSY services compared with those who did not. Moreover, infant deaths among women who received JSY benefits (1.4%) was significantly lower compared with those who did not receive JSY benefits (10.4%).

### Factors associated with receipt of JSY benefits

Of the 1,806 women, only 932 (51.6%) (95% CI: 49.3–53.9) reported having received JSY benefits. Results of logistic regression analyses are presented in Table 3. In the adjusted model, pregnant woman's spouse having no formal or primary education, having ≤4,000 INR income, not receiving visits from ASHA, and delivery at a subcenter or primary health care center or district hospital were significant determinants of JSY receipt of services. Pregnant women's spouse without any formal education had 1.4 times increased odds of receiving JSY (AOR: 1.35; 95% CI: 1.01–1.80) compared with those with more than primary education (>8 years). Moreover, spouses who completed primary education (8 years) had 1.3 times increased odds of receiving JSY (AOR: 1.31; 95% CI: 1.05–1.64) compared with those with more than primary education (>8 years). Women who had a monthly household income of ≤4,000 INR had 1.5 times increased odds of receiving JSY (AOR: 1.47; 95% CI: 1.04–2.09) compared with women with a monthly household income of ≥10,000.

**Table 3.** Sociodemographic Factors, Program Initiatives, and Birth Outcomes by Receipt of *Janani Suraksha Yojana* Benefits

Variables	Receipt of JSY benefits	
	Unadjusted OR (95% CI)	AOR^*^ (95% CI)
Sociodemographic factors		
Age (mean, SD)	***0.97 (0.94–0.99)***	0.98 (0.94–1.01)
Pregnant woman's education (in years)		
No education	0.71 (0.48–1.05)	0.71 (0.46–1.09)
Primary (1–8)	0.97 (0.79–1.18)	0.90 (0.73–1.12)
More than primary (>8)	Ref.	Ref.
Partner's education (in years)		
No education	1.23 (0.95–1.60)	***1.35 (1.01–1.80)***
Primary (1–8)	***1.29 (1.05–1.59)***	***1.31 (1.05–1.64)***
More than primary (>8)	Ref.	Ref.
Total monthly household income (in INR)		
≤4,000	1.38 (1.00–1.91)	***1.47 (1.04–2.09)***
4,001–10,000	1.29 (0.94–1.76)	***1.40 (1.01–1.95)***
>10,000	Ref.	Ref.
Microeconomic self-help group membership		
No	0.85 (0.71–1.02)	0.82 (0.68–1.01)
Yes	Ref.	Ref.
Programs		
ASHA visits during pregnancy		
Regularly (once a month)	***1.38 (1.07–1.79)***	1.21 (0.89–1.67)
Occasionally (once in 2 months)	0.85 (0.56–1.31)	1.18 (0.68–2.06)
Rarely (once in 3 months)	***4.48 (2.07–9.72)***	***3.55 (1.55–8.51)***
Never	Ref.	Ref.
ASHA accompanying woman to her ANC visits		
Regularly (once a month)	1.02 (0.84–1.24)	1.00 (0.79–1.27)
Occasionally (once in 2 months)	0.60 (0.42–0.85)	0.73 (0.45–1.17)
Rarely (once in 3 months)	***3.13 (1.57–6.23)***	***2.45 (1.17–5.13)***
Never	Ref.	Ref.
ASHA arranged for ambulance during labor		
No	***0.59 (0.49–0.71)***	***0.67 (0.55–0.82)***
Yes	Ref.	Ref.
Pregnancy outcomes		
Gravida		
Primigravida	0.98 (0.81–1.19)	1.02 (0.82–1.26)
Multigravida	Ref.	Ref.
Antenatal checkups		
≤4 antenatal checkups	***2.19 (1.13–4.24)***	1.91 (0.96–3.78)
5–9 antenatal checkups	1.71 (0.84–3.50)	1.62 (0.77–3.40)
10 and more antenatal visits	Ref.	Ref.
Delivery place		
Home	0.43 (0.17–1.06)	0.44 (0.17–1.13)
Public institution	1.22 (1.00–1.48)	***1.23 (1.01–1.51)***
Private institution	Ref.	Ref.

The bold/italic highlights the statistically significant values.

^*^ The final model was adjusted for age, pregnant woman's education, partner's education, total monthly household income, microeconomic self-help group, ASHA visits during pregnancy, ASHA accompanying women for ANC visits, ASHA arrangement for ambulance during labor, gravida, birth weight, antenatal checkups, and delivery place.

AOR, adjusted odds ratio; CI, confidence intervals; OR, odds ratio.

Women who received rare visits (once in 3 months) from ASHA during pregnancy had >3.6 times the odds of receiving JSY benefits compared with those with no visits at all (AOR: 3.55; 95% CI: 1.55–8.51). However, women who received either regular visits from ASHA during pregnancy or occasional visits compared with no visits were not statistically significant. More specifically in terms of ANC visits, women who were rarely accompanied (once in 3 months) by ASHA during their ANC visits had nearly 2.5 times increased odds of receiving JSY benefits (AOR: 2.45; 95% CI: 1.17–5.13) compared with those who never had any ASHA visits during ANC. Women who delivered in a subcenter, primary health care center, or district health had 1.2 times increased odds of receiving JSY (AOR: 1.23; 95% CI: 1.05–1.51) compared with women who delivered at a maternity home/private nursing hospital. However, women who did not receive any planned ambulance transportation service had 0.33 times decreased odds of receiving JSY benefits (AOR: 0.67; 95% CI: 0.55–0.82) compared to women who received planned ambulance transportation.

## Discussion

Given that JSY implementation occurred in 2005, more than a decade ago, there were vast disparities that continue to exist in receipt of JSY benefits. The present study indicated that only 50% of marginalized and rural pregnant women benefited from the JSY (conditional cash transfer program), highlighting the inadequacies of the program. Moreover, the receipt of JSY services is much lower than their rural counterparts in other states.^12^ While most of the Indian population (69%) reside in rural areas,^19^ it is imperative to impart JSY knowledge to women to access and utilize health care to promote healthy and safe births, and reduce maternal and infant mortality. The findings indicate that low-income women, with spousal education being less than primary education, were significant determinants of being a recipient of JSY services. It is worth noting that nearly half (46.6%) of those who earned ≤4,000 INR failed to receive JSY benefits highlighting the inherent need that the program did not specifically target the low-income pregnant women for whom the services were intended and favored those within a higher income bracket. More importantly, our findings confirmed that JSY benefits are yet to impact the marginalized women of lower income and educational status, which remains supported by previous studies.^4,6,20^ While JSY has catered to improving institutional delivery and prevent adverse birth outcomes, this analysis shows that among those pregnant women who did not receive JSY, ∼10% reported infant deaths and 14.5% delivered infants who had low birth weight (<2,500 g) and 1.9% had home delivery compared with those who received JSY services.

While it is heartening to report that the majority of the births of JSY beneficiaries occurred in institutional facilities. Similar to other reported studies, our study also showed that delivery in a governmental institution (subcenter, primary health center, or district health center) was significantly associated with the receipt of JSY benefits.^7,13,21,22^ Almost less than half (44.9%) of the women who received JSY payments delivered within a public facility. However, the majority of women preferred delivery within a maternity home or private nursing. It is crucial to promote institutional delivery since home deliveries can further lead to complications and may lead to severe maternal morbidity or mortality. One possible explanation for preference may have been the quality of obstetric care and services offered in private facilities compared with government facilities, leading to preference for private facilities for their deliveries.^23^ These findings are contrary to the 2015/2016 National Family Health Survey findings in Karnataka, which noted that ∼94% of the births in the previous 5 years occurred in a health facility, of which a majority were in government institutions.^15^

The Government of India also proposed that JSY be facilitated by ASHA workers to provide linkage to health care services during pregnancy. Our study findings showed that JSY receipt of services was not directly connected through ASHA workers, since there was still a majority of those who did not receive JSY benefits, but still had regular or occasional interaction with ASHA workers. In addition, there was limited interaction with health care workers and social workers such as ASHA, specifically among women who did not receive JSY benefits. Similar studies by Dongre and Kapur^21^ and Sidney et al.^24^ indicated that ASHA had limited involvement, with only 28% being involved during delivery or postdelivery. Although ASHA serves the central role of connecting pregnant women to ANC, identifying institutional delivery site, arranging transport, and staying with pregnant women during delivery until time of discharge, the government needs to enact an accountability measure to ensure that the women are indeed well-connected to ASHA to receive JSY services.

There were several inconsistencies with the lack of uniform payment and timely disbursement of the funding with the JSY benefits. Many women (45.5%) reported having received 1,500 INR, while a small number of women (1.5%) had received 500 INR and another 37.7% reported having received 700 INR. Since Karnataka is assigned as a high-performing state, the government allocates 700 INR to mothers residing in rural areas and 600 INR to mothers residing in urban areas. An underlying reason is that the JSY scheme in Mysore District allocated 700 INR for vaginal delivery and 1,500 INR for cesarean section delivery. However, the remaining women who provided some form, but not all forms, of documentation received 500 INR. According to Doke et al., nearly 20% of the women faced problems in obtaining BPL certificate of documentation for JSY benefits.^25^

Almost 65% of the women received the JSY payments after 30 days of delivery. This is contrary to prior studies which noted that the majority of the women received JSY payments within 15 days. It is imperative to understand the inequitable access of payments to the pregnant women. It could be the underlying unpaid work burden that many rural families face. Rural regions in the state of Karnataka have worsened maternal and child health outcomes.^15^ In 2016 alone, rural regions of the state had infant mortality rate of 33 per 1,000 live births compared with the overall infant mortality rate of 28 per 1,000 live births for the state of Karnataka.^15^ This warrants further examination for ensuring that rural regions in high-performing states are still compensated accordingly since they continue to encounter major barriers in access to good-quality obstetric care and services.

This study was among the first to examine the receipt of JSY benefits among rural pregnant women in the South Indian State of Karnataka. The strengths of this study are that it was a prospective cohort study with a large sample size from a rural region within India. In addition, the pregnant women were followed up after birth making it a rich longitudinal data set. Since the questionnaire about JSY was collected in real time and was self-reported, it is more objective. Despite these strengths, there were some limitations. One major limitation of the study was that it did not include a survey question on the ownership of the BPL card as a measure indicator for eligibility of JSY benefits. Therefore, it is difficult to examine if the discrepancies seen are linked to lack of proper documentation, or women with higher as well as lower income levels making them ineligible for JSY benefits.

Furthermore, given that participants were selected from women residing in the Mysore Subdistrict, which is predominantly rural, the findings may not be generalizable to other populations. Due to the self-reporting nature of some questions, it is possible that there may be some degree of information bias. Overall, the study confirmed that allocation of JSY benefits needs to reach the marginalized and vulnerable women in rural Indian states.

## Conclusions

Although the JSY program has been operational since 2005, our study findings show discrepancies in the receipt of JSY scheme in the rural Mysore Subdistrict, India. Therefore, there is an urgent need for targeted education and expansion of comprehensive antenatal services to provide the JSY scheme to rural pregnant women in India and best serve these disproportionately affected women.

## Abbreviations Used

ANC: antenatal care
ANM: auxiliary nurse midwives
AOR: adjusted odds ratio
ASHA: Accredited Social Health Activists
BPL: below poverty line
CI: confidence interval
INR: Indian Rupees
JSY: *Janani Suraksha Yojana*
MMR: maternal mortality ratio
OR: odds ratio
SCIL: Saving Children Improving Lives
SD: standard deviation
USD: U.S. Dollar
